# Deletion of astrocytic BMAL1 results in metabolic imbalance and shorter lifespan in mice

**DOI:** 10.1002/glia.23764

**Published:** 2019-12-13

**Authors:** Olga Barca‐Mayo, Arjen J. Boender, Andrea Armirotti, Davide De Pietri Tonelli

**Affiliations:** ^1^ Neurobiology of miRNA lab Fondazione Istituto Italiano di Tecnologia Genoa Italy; ^2^ Neuromodulation of Cortical and Subcortical Circuits Lab Fondazione Istituto Italiano di Tecnologia Genoa Italy; ^3^ D3 PharmaChemistry Fondazione Istituto Italiano di Tecnologia Genoa Italy

**Keywords:** astrocytes, circadian clock, GABA signaling, glutamate, lifespan, metabolism

## Abstract

Disruption of the circadian cycle is strongly associated with metabolic imbalance and reduced longevity in humans. Also, rodent models of circadian arrhythmia, such as the constitutive knockout of the clock gene *Bmal1*, leads to metabolic disturbances and early death. Although astrocyte clock regulates molecular and behavioral circadian rhythms, its involvement in the regulation of energy balance and lifespan is unknown. Here, we show that astrocyte‐specific deletion of *Bmal1* is sufficient to alter energy balance, glucose homeostasis, and reduce lifespan. Mutant animals displayed impaired hypothalamic molecular clock, age‐dependent astrogliosis, apoptosis of hypothalamic astrocytes, and increased glutamate and GABA levels. Importantly, modulation of GABAA‐receptor signaling completely restored glutamate levels, delayed the reactive gliosis as well as the metabolic phenotypes and expanded the lifespan of the mutants. Our results demonstrate that the astrocytic clock can influence many aspects of brain function and neurological disease and suggest astrocytes and GABAA receptor as pharmacological targets to prevent the metabolic dysfunctions and shortened lifespan associated with alterations of circadian rhythms.

## Graphical abstract

- Astrocyte clock controls energy balance, glucose homeostasis and longevity in mice.
- Modulation of GABAA‐receptor signaling delays the metabolic phenotype and expanded the life span of mice with astrocytic deletion of BMAL1.

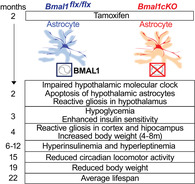

## 1 INTRODUCTION

The circadian clock is an endogenous, self‐sustaining oscillator that operates with a periodicity of 24 hr to maintain proper rhythms in gene expression, physiology, and behavior (reviewed in Hastings, Maywood, & Reddy, 2008). As evidenced by individuals working in the night or rotating shifts and in rodent models of circadian arrhythmia, disruption of the circadian cycle is strongly associated with metabolic imbalance and reduced longevity (reviewed in Chaudhari, Gupta, Makwana, & Kondratov, 2017; Karlsson, Knutsson, Lindahl, & Alfredsson, 2003; Parkes, 2002; van Amelsvoort, Schouten, & Kokn, 1999).

The timekeeping system includes cellular autonomous clocks that are entrained by hormonal and neuronal signals from a central pacemaker: the suprachiasmatic nuclei (SCN), located in the hypothalamus (Moore & Eichler, 1972; Stephan & Zucker, 1972). The molecular mechanisms driving circadian oscillations involve rhythmic and self‐sustained transcriptional–translational feedback loops of clock genes/proteins. The E‐box specific transcription factors BMAL1 (Brain and muscle Arnt‐like protein‐1) and CLOCK (Circadian locomotor output cycles kaput) are the positive limb of this oscillatory mechanism, which heterodimerize to activate transcription of the repressors *Period* (*Per1/2/3*) and *Cryptochrome* genes (*Cry1/2*; van der Horst et al., 1999; Zheng et al., 2001). The negative loop of the molecular clock is constituted by PER/CRY heterocomplex that, upon accumulation, lead to degradation of BMAL1/CLOCK dimers thus inhibiting their own transcription (Kume et al., 1999). CLOCK/BMAL1 drives rhythmic expression of clock‐controlled genes, which regulate many physiological processes including major components of energy homeostasis such as feeding behavior, locomotor activity, sleep–wake cycle and glucose metabolism (reviewed in Marcheva, Ramsey, Affinati, & Bass, 2009; Richards & Gumz, 2013). Indeed, the absence of BMAL1 in mice results in a loss of circadian rhythms, alteration in energy balance, acceleration of aging and shortened lifespan (Bunger et al., 2005; Kondratov, Kondratova, Gorbacheva, Vykhovanets, & Antoch, 2006; Lamia, Storch, & Weitz, 2008; Lee, Donehower, Herron, Moore, & Fu, 2006; Marcheva et al., 2010; Rudic et al., 2004; Shi, Ansari, McGuinness, Wasserman, & Johnson, 2006).

In mammals, neurons of the SCN have been classically considered as master pacemaker cells, controlling daily rhythms of physiology and behavior and coordinating the circadian programs of peripheral tissues (Yoo et al., 2004). However, several evidence have recently challenged this neurocentric view of the timekeeping system by uncovering that astrocytes autonomously function as a central circadian clock regulating molecular and behavioral circadian rhythms (Barca‐Mayo et al., 2017; Brancaccio et al., 2019; Brancaccio, Patton, Chesham, Maywood, & Hastings, 2017; Tso et al., 2017). In light of this new scenario, we hypothesize that astrocytic clock might have a crucial contribution to the circadian control of metabolism and lifespan.

Here, we provide evidence that adult disruption of the astrocytic clock, via genetic deletion of BMAL1, is sufficient to lead to the metabolic and age‐associated dysfunctions of constitutive *Bmal1* knockout animals. Specifically, the deletion of BMAL1 in astrocytes impairs the hypothalamic circadian function and leads to increased GABA and glutamate levels, age‐dependent astrogliosis and apoptosis of hypothalamic astrocytes, altered glucose homeostasis, altered growth curves, and reduced lifespan. Remarkably, these phenotypes were delayed upon inhibition of GABAA receptor signaling. Our results demonstrate that the astrocytic molecular clock can influence many aspects of brain function and neurological disease and suggest astrocytes and GABAA receptor as targets for chronotherapies to prevent dysfunctions associated with chronic or acute alterations of circadian rhythms.

## 2 MATERIALS AND METHODS

### 2.1 Animals

All experiments and procedures were approved by the Italian Ministry of Health (Permit No. 214/2015‐PR) and the local Animal Use Committee, and were conducted in accordance with the Guide for the Care and Use of Laboratory Animals of the European Community Council Directives and of Italian Ministry of Health. Bmal1^flox/flox^ mice (Jackson Laboratory Stock 007668, B6.129S4 (Cg) Arntltm1Weit/J, RRID: IMSR_JAX:007668) were crossed with Glast (Glutamate aspartate transporter) creERT2^+/−^ mouse line (RRID: MGI: 3830092). *Bmal1* conditional knockout mice were generated by treating 6‐ to 8‐weeks‐old male *Glast‐CreERT2* ^+/−^:*Bmal1* ^*f*lox/flox^ with tamoxifen (TM; *Bmal1cKO*), as previously reported (Barca‐Mayo et al., 2017). TM‐treated *Bmal1* ^flox/flox^ male littermates served as controls. Note that the functional glutamate uptake, live span, energy balance, and glucose homeostasis is unaffected in Glast‐CreERT2^+/−^ mice (García‐Cáceres et al., 2016; Saab et al., 2012). Mice were housed with ad libitum access to food and water, and kept on a 12 hr (8 a.m. to 8 p.m.) light–dark cycle, in a room, maintained at 21°C at the animal facility of the Istituto Italiano di Tecnologia (IIT), Genoa, Italy. Pentylenetetrazole (PTZ) treatment of control and *Bmal1cKO* mice was performed as previously reported (Barca‐Mayo et al., 2017). Feeding behaviors were examined in mice housed in individual cages by measuring the daily, the daytime (immediately after the onset of the light period (8:00 a.m., Zeitgeber [ZT0]), and nighttime (immediately before the onset of the dark period [8:00 p.m., ZT12]), food intake. Specifically, food intake was assessed by subtracting the amount of food remaining in the cages from the amount provided to the animals the previous day or daytime (ZT0 and ZT12). Food spillage was minimal, and was assessed by visual inspection and accounted for when necessary. Averages represent the intake over 2 consecutive days.

### 2.2 Locomotor activity

Two to three or 15 months after TM treatment, male *Bmal1cKO* and control mice were single‐housed in cages equipped with running wheels (ENV‐044; Med Associates, Inc). Mice were adapted to the wheel for 3 days in standard light–dark cycles (12:12 hr, lights on at 8 a.m., ZT0). Running wheel activity monitoring started under these conditions during the next 3–5 days. Running wheel activity was recorded in 5 min (min) bins by Wheel Manager software (SOF‐860; Med Associates, Inc) as previously reported (Barca‐Mayo et al., 2017).

### 2.3 Operant conditioning

Two months after TM treatment, male control and *Bmal1cKO* mice were subjected to a restricted feeding schedule where the access to standard lab chow (Special Diet Services, UK) was restricted from 16:00 to 18:00 (i.e., ZT8 and ZT10, respectively). Behavioral experiments started 3 days after the restricted feeding schedule and were conducted between 14:00 and 16:00. The behavioral training took place in operant chambers (17.8 cm × 15.2 cm × 18.4 cm) in which two holes were placed on either side of a food magazine (Med Associates, St. Albans, VT). Sucrose pellets (SP, TestDiet, Indianapolis, IN) were delivered into the food magazine when the mouse nose poked into the “active” hole (ANP), whereas a poke into the “inactive” hole had no consequence. Initial nose poke training consisted of three daily sessions of fixed ratio (FR) schedule (FR‐1), in which every active nose poke was rewarded. After the FR1 sessions, mice were trained on a FR‐5 and FR‐25 schedules, in which every fifth or 25th active nose poke led to SP delivery, respectively. After three daily FR‐5 sessions and three daily FR‐25 schedules, mice were trained under a progressive ratio (PR) schedule for 3 days. In the PR‐schedule, the number of ANP required to obtain SP is increased with each completed trial (ANP  =  5 × e^0.2SP^) so each successive SP required more ANP and the amount of ANP reflected the effort that was invested in the task.

### 2.4 Glucose tolerance test

Control and *Bmal1cKO* mice (at the age and time point specified) were fasted for 16 hr before the glucose tolerance test. Blood was obtained from a tail cut and was assessed for fasting glucose levels using an OneTouch Ultra 2 (LifeScan, Johnson & Johnson) glucometer. Mice then received a glucose solution (2 g/kg body weight) delivered by intraperitoneal injection. At 15, 30, 60, 90, and 120 min after the administration, dried blood was quickly removed from the tail wound and fresh blood was collected again to measure the glucose concentration.

### 2.5 Determination of serum leptin, insulin, corticosterone, and glucagon

Leptin, insulin, corticosterone, and glucagon serum levels were determined by ELISA using reagents kits and methods provided by Merck‐Millipore (insulin, EZRMI‐13K; leptin, EZML‐82K), Enzo Life Sciences (corticosterone, ADI‐900‐097) and Sigma **(**Glucagon, RAB0202).

### 2.6 Determination of GABA/glutamate levels in cerebrospinal fluid

GABA was quantified in mouse cerebrospinal fluid (CSF) samples, collected from the cisterna magna, by UPLC‐MS/MS (Ultra Performance Liquid Chromatography–Tandem mass spectrometry) as we previously described (Barca‐Mayo et al., 2017). Glutamate was extracted from CSF by precipitation with acetonitrile spiked with deuterated Glutamate (D5) as an internal standard (Sigma Aldrich). The analytes were then separated by HILIC chromatography (Hydrophilic Interaction Liquid Chromatography) using a BEH HILIC 2.1X100 mm. column and a short gradient of water in acetonitrile (5–40% in 2 min), with the eluent added with formic acid to a final 0.1% v/v, flow rate was kept at 0.45 ml/min. Glutamate was quantified on a Xevo TQ‐MS instrument operating in electrospray, positive ion mode and following the MRM (Multiple Reaction Monitoring) transitions. Both the column and the UPLC‐MS/MS systems were purchased from Waters Inc. (Milford). Glutamate quantification was performed using a standard calibration curve prepared by serial dilution in artificial CSF and extracted along with the samples. The investigators were blinded to group allocation during experiments.

### 2.7 Immunofluorescence

Mice were administered ketamine/xylazine (150 mg/kg, 10 mg/kg, respectively) and transcardially perfused with ice‐cold PBS followed by ice‐cold 4% PFA in PBS. Brains were post‐fixed overnight in 4% PFA in PBS and 30 μm slices were prepared Cryostat (Leica). Slices were permeabilized with 0.3% Triton X‐100 in PBS, blocked with 10% goat serum in PBS, and incubated at 4°C overnight with the primary antibody mouse anti‐Glial Fibrillary Acidic Protein (GFAP) 1:1,000 dilution (Sigma, G3893); rabbit anti‐BMAL1, dilution 1:200 (Abcam, ab93806); mouse anti‐S100 calcium‐binding protein β (S100β), 1:1,000 dilution (Sigma, AMAB91038); rabbit anti‐active CASPASE 3, 1:1,000 dilution (Cell Signaling Technology, 9579); mouse anti‐RNA Binding Fox‐1 Homolog 2 (FOX2) 1:500 dilution (Abcam, ab57154); rabbit anti‐KI67 1:200 dilution (Thermo Scientific MA5‐14520). The following day, sections were extensively washed, and incubated for 2 hr with goat anti‐rabbit or anti‐mouse Alexa‐488 or Alexa‐546 secondary antibodies (1:1,000 dilution). Slices were then washed, mounted with Prolong Gold and imaged with an inverted laser scanning confocal microscope (TCS SP5 microscope using a 20× or 40× objective, Leica Microsystems). Quantification and analysis were performed with ImageJ software (Wayne Rasband, NIH), by outlining the hypothalamus from the 4′,6‐diamidino‐2‐phenylindole (DAPI)‐stained image and using this template to measure the relative intensity of the immunostaining. When more than one section was analyzed from each animal, the mean of the measures from consecutive sections was used for that individual.

### 2.8 RNA isolation and quantitative real‐time RT‐PCR

Total RNA was extracted from hypothalamus using TRIzol reagent following the manufacturer's instructions. RNA was further cleaned using an RNeasy Mini Kit. cDNA was obtained by reverse transcription of 0.5 μg of total mRNA using the ImProm‐II™ Reverse Transcription System following the manufacturer's instructions. Real‐time RT‐PCR was done using the ABI PRISM.7900 (Applied Biosystems). For a 15 μl reaction, 9 ng of cDNA template was mixed with the primers to a final concentration of 200 nM and mixed with 7.5 μl of 2× QuantiFast SYBR Green PCR Master Mix. The reactions were done in duplicates using the following conditions: 5 min at 95°C followed by 40 cycles of 10 s at 95°C, 30 s at 60°C, and 1 min at 70°C. Glyceraldehyde 3‐phosphate dehydrogenase (GAPDH) or beta‐actin transcripts were used as reference controls.

### 2.9 Statistical analysis

Statistical parameters including the exact value of *n*, and precision measures (mean ± SEM) and statistical significance are reported in the figures and figure legends. All statistical tests were two‐sided. A log‐rank test was used for survival curve analysis. Other statistical comparisons were done by Student's paired *t* test, or two‐way ANOVA with a post hoc Bonferroni. Data were checked for normality and equal variances between groups. Statistical significance of the rhythmic expression was determined by Cosinor analysis as previously reported (Barca‐Mayo et al., 2017). The cutoff for significance was *p < .05, **p < .01, ***p < .001, and ****p < .0001. Statistical analysis was performed with GraphPad PRISM 6 software.

## 3 RESULTS

### 3.1 Deletion of BMAL1 in astrocytes leads to early death, altered body weight, and glucose homeostasis

**Figure 1:**
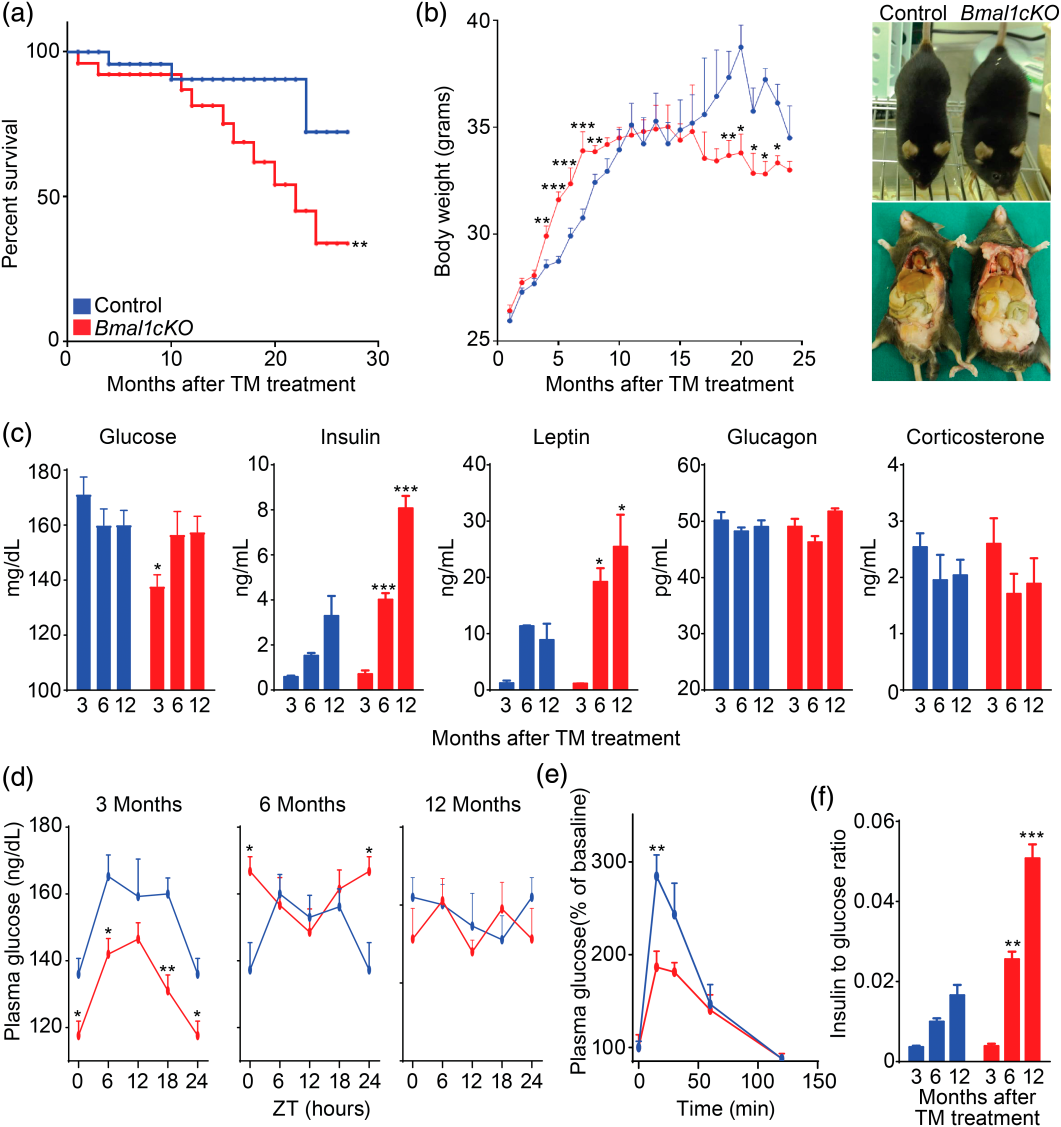
Loss of *Bmal1* in astrocytes leads to early death, altered body weight and glucose homeostasis. (a) Kaplan‐Meyer survival curve of control and *Bmal1cKO* mice (*n*  =  24 and *n* = 16, respectively, Log rank test, **p < .01). (b) Left panel, age‐dependent changes in body weight of control and *Bmal1cKO* males. Data are represented as mean ± SEM (*n*  =  18, paired *t*‐test, *p < .05, **p < .01, and ***p < .001 vs. control animals). Right panel, gross appearance of control and *Bmal1cKO* mice 4 months after TM treatment, showing increased fat mass in the mutants. (c) Blood glucose, insulin, leptin, glucagon, and corticosterone levels in control and *Bmal1cKO* mice at 3, 6, and 12 months after TM treatment (ZT6). Data are represented as mean ± SEM (*n*  =  8, paired *t*‐test, *p < .05 and ***p < .001 vs. control animals). (d) Blood glucose in control and *Bmal1cKO* animals after 3, 6, and 12 months of TM treatment. Data are represented as mean ± SEM (*n*  =  8, paired *t*‐test, *p < .05 and **p < .01 vs. control animals). (e) Glucose tolerance test in control and *Bmal1cKO* mice at 3 months after TM treatment performed at ZT10. Data are represented as mean ± SEM (*n* = 5–6, paired *t*‐test, **p < .01 vs. control animals). (*f*) Insulin to glucose ratio of control and *Bmal1cKO* animals at 3, 6, and 12 months of TM treatment (ZT6). Data are represented as mean ± SEM (*n*  =  5–6, paired *t*‐test, **p < .01 and ***p < .001 vs. control animals) [Color figure can be viewed at wileyonlinelibrary.com]

To investigate the contribution of astrocyte clock in the regulation of lifespan and energy balance, we genetically deleted *Bmal1* in astrocytes expressing GLAST by crossing *Bmal1* ^flox/flox^ mice with a tamoxifen (TM) inducible knock‐in *Glast‐CreERT2* ^+/−^ deletor mouse line (Mori et al., 2006), here referred to as *Bmal1cKO*. We administered TM to 6‐ to 8‐weeks‐old *Bmal1cKO* male mice and controls (*Bmal1* ^*flox/flox*^), an approach that we have previously shown to achieve astrocyte‐ and time‐specific deletion of *Bmal1*, while avoiding functional abnormalities or compensations that might occur during development (Barca‐Mayo et al., 2017; Mori et al., 2006). Following TM administration, we kept mice in 12‐hr: 12‐hr light–dark cycles and allowed them to feed ad libitum with regular diet. We found that the lifespan of *Bmal1cKO* animals was significantly reduced compared to controls (Figure 1a). Specifically, most mutants died between 20 and 25 months of age (average of lifespan, 22 months after TM treatment; Figure 1a). *Bmal1cKO* mice were significantly heavier than control animals from 4 until 8 months after TM treatment, with increased adipose tissue mass, and their body weight progressively decreased after 19 months of TM treatment (Figure 1b). Similarly, higher body weight and greater adipose tissue mass than control animals were previously found in constitutive *Bmal1* ^*−/−*^ mice at 1–2 months of age, followed by a steady decrease (Table S1), a phenotype that was regarded as hallmark of premature aging (Bunger et al., 2005; Kondratov et al., 2006; Lamia et al., 2008; Lee et al., 2006). Therefore, we concluded that the deletion of BMAL1 in astrocytes is sufficient to shorten the lifespan and alter the growth curves as previously observed in *Bmal1 −/−* mice (Table S1).

To determine whether the altered growth curves of *Bmal1cKO* animals were associated with changes in metabolic markers, we analyzed blood levels of glucose, insulin, glucagon, leptin, and corticosterone in the mutants and controls at 3, 6, and 12 months after TM treatment, corresponding to the onset of increased weight gain (i.e., pre‐obese stage), transient obesity and to the period in which bodyweight of mutant mice was indistinguishable from control animals, respectively. We found that *Bmal1cKO* animals had hypoglycemia at 3 months after TM treatment and hyperinsulinemia and hyperleptinemia at 6 and 12 months after TM treatment (Figure 1c). These alterations were not due to changes in glucocorticoid production, because levels of corticosterone were indistinguishable between *Bmal1cKO* and controls (Figure 1c). Similarly, no differences were found in glucagon levels between both groups of mice (Figure 1c). Remarkably, at 3 months after TM treatment, *Bmal1cKOs* had lower levels of glucose throughout the day (Figure 1d). However, at later stages no differences were found in blood glucose between mutants and control at different times of the day, with the exception of significantly higher levels in the mutants at ZT0, 6 months after TM treatment (Figure 1d).

To gain insights into the hypoglycemia of *Bmal1cKOs* at the pre‐obese stage (i.e., 3 months after TM treatment), we performed a glucose tolerance test. Interestingly, mutant mice showed a blunted elevation of blood glucose compared to controls (Figure 1e). The mean peak increase in blood glucose level (normalized as a percentage of the initial level) of *Bmal1cKOs* was 34.47% lower than that of controls (186.43% ± 17.25 vs. 284.49% ± 22.89%, paired *t* test, *p* = .01). These results indicated enhanced insulin sensitivity in *Bmal1cKOs* and were consistent with previous findings in constitutive *Bmal1−/−* and *ClockΔ19* animals (Table S1; Lamia et al., 2008; Marcheva et al., 2010; Rudic et al., 2004). Defective glucose regulation typically worsens with age (reviewed in Neubauer & Kulkarni, 2006). Indeed, *Bmal1cKOs* showed significantly higher levels of insulin at 6 and 12 months after TM treatment, leading to increased insulin to glucose ratio (Figure 1f). This indicates that *Bmal1cKOs* developed age‐dependent insulin resistance, likely associated with their increased body weight.

Altogether, our results indicate that adult deletion of *Bmal1* in a subpopulation of astrocytes is sufficient reduce lifespan, alter body weight and glucose homeostasis as previously reported in constitutive *Bmal1*−/− animals (Table S1; Bunger et al., 2005; Kondratov et al., 2006; Lamia et al., 2008; Lee et al., 2006; Marcheva et al., 2010; Rudic et al., 2004; Shi et al., 2006).

### 3.2 *Bmal1cKO* mice showed increased food intake with no alterations in the brain reward systems

**Figure 2:**
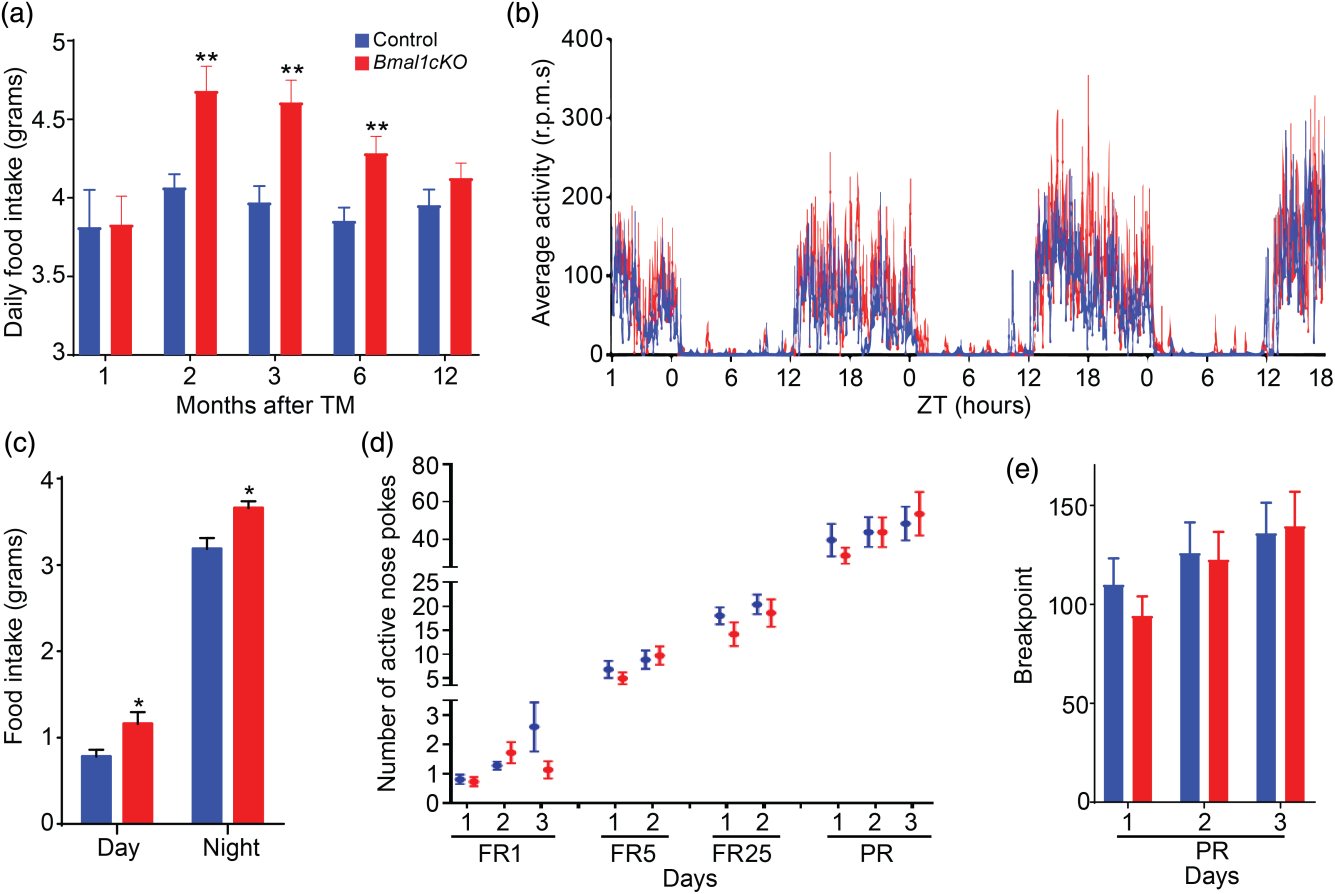
*Bmal1cKO* mice showed increased food intake with no alterations in the brain reward systems. (a) Daily food intake was determined in control and *Bmal1cKO* mice after 1, 2, 3, 6, and 12 months of TM treatment. Animals were maintained in 12 hr:12 hr light–dark cycles and fed ad libitum with a standard mouse chow. Data are represented as mean ± SEM (*n* = 8, paired *t*‐test, **p < .01 vs. control animals). (b) Activity waveforms for control (*n* = 8) and *Bmal1cKO* (*n* = 7) mice, 2 months after TM treatment, in 12 hr: 12 hr light–dark cycles. Activity counts are expressed as the average amount of activity in 5 min bins. Data plotted is given in ZT, such that ZT0 = lights on. The value expresses the means + SEM. (c) Food intake was determined in control and *Bmal1cKO* mice after 2 months of TM treatment. Animals were maintained in 12 hr:12 hr light–dark cycles and fed ad libitum with a standard mouse chow. Data are represented as mean ± SEM (*n* = 8, paired *t*‐test, *p < .05 vs. control animals). (d) Number of nose spokes performed by control and *Bmal1cKO* mice, 2 months after TM treatment, during fixed ratio (FR), and progressive ratio (PR) sessions in the operant conditioning test. Data are represented as mean ± SEM (*n* = 4 for controls and *n* = 11 for *Bmal1cKOs*). (e) Mean ± SEM breakpoints in control (*n* = 4) and *Bmal1cKO* (*n* = 11) mice, 2 months after TM treatment, in the operant conditioning test [Color figure can be viewed at wileyonlinelibrary.com]

Bodyweight results from the homeostatic regulation to balance energy intake and energy expenditure. We monitored, at different times after TM treatment, the daily food intake of control and mutant animals that were kept in normal light–dark cycles. We observed that 1 month after TM treatment, *Bmal1cKO* animals had comparable food intake than control mice (Figure 2a). However, from 2 to 6 months after TM treatment, *Bmal1cKOs* had significantly increased food intake than controls (Figure 2a), therefore preceding their increase in the body weight (observed 4 months after TM treatment, Figure 1b) and their insulin resistance (detected 6 months after TM treatment, Figure 1f). Twelve months after TM treatment, when the bodyweight of mutant mice was indistinguishable from control animals (Figure 1b), the mutants showed no differences in the food intake compared to controls (Figure 2a). On the other hand, we found no differences in the daily activity between control and *Bmal1cKO* mice 2 months after TM treatment (Figure 2b), consistent with our previous observations (Barca‐Mayo et al., 2017). However, at this pre‐obese stage, *Bmal1cKO* animals had abnormal feeding behavior as shown by their significantly increased food intake at both day and night times (Figure 2c). It was shown that the metabolic dysfunctions and obesity of mouse models with genetic disruptions in core clock genes such as *Clock*, are directly linked to disturbed feeding rhythms and/or excessive daytime feeding (Turek et al., 2005). Therefore, we postulate that the altered feeding pattern of *Bmal1cKO* mice at the pre‐obese stage results in obesity and insulin resistance at later stages.

The main brain region regulating homeostatic food intake is the arcuate nucleus (ARC) of the hypothalamus through its connections with other hypothalamic nuclei and extra‐hypothalamic brain areas (reviewed in Coll, Farooqi, & O'Rahilly, 2007 and in Blouet & Schwartz, 2010; Dietrich & Horvath, 2009; van Vliet‐Ostaptchouk, Hofker, van der Schouw, Wijmenga, & Onl‐Moret, 2009). However, other brain circuits involved in the rewarding effects of food such as several limbic (nucleus accumbens, amygdala and hippocampus) and cortical brain regions (orbitofrontal cortex, cingulate gyrus, and insula) are also implicated in hedonic‐driven food consumption and obesity (reviewed in Coll et al., 2007 and in Belgardt, Okamura, & Brüning, 2009; Goldstone, 2006; Rolls, 2008). To address whether the increased food intake of the mutant was due to an alteration in hedonic system, we subjected to control and *Bmal1cKO* mice (after 2 months of TM treatment) to a progressive‐ratio schedule of food‐pellet reinforcement, a commonly used measure of reward strength (Hodos, 1961, 1963). Animals were trained to nose‐poke in an active hole to get a food reward under FR1, FR5, and FR25 schedules. No significant differences were detected between *Bmal1cKO* and control mice in the acquisition of the task (Figure 2d). Moreover, in the PR session, in which mice had to nose‐poke an increasing number of times to get the same reward (breakpoint), *Bmal1cKO* mice poked as control animals and earned the same rewards (Figure 2e). This result suggests that increased food intake in *Bmal1cKO* mice is due to an alteration in the hypothalamic homeostatic system and less likely to hedonic search for food.

Astrocytes have been recently postulated as key contributors to energy balance regulation and obesity due to the discovery of hypothalamic inflammation and gliosis, particularly in the ARC nucleus, in obese rodents and humans (reviewed in McNay, Briançon, Kokoeva, Maratos‐Flier, & Flier, 2012; Thaler et al., 2012; Valdearcos, Xu, & Koliwad, 2015). Remarkably, it was reported that BMAL1 is a potent regulator of astrocyte activation or gliosis (Lananna et al., 2018; Musiek et al., 2013). Therefore, we hypothesize that BMAL1 may connect dysregulation of circadian function to hypothalamic astrogliosis leading to the increased body weight and metabolic alteration of *Bmal1cKO* mice.

### 3.3 BMAL1 deletion in astrocytes globally impairs the hypothalamic molecular clock

**Figure 3:**
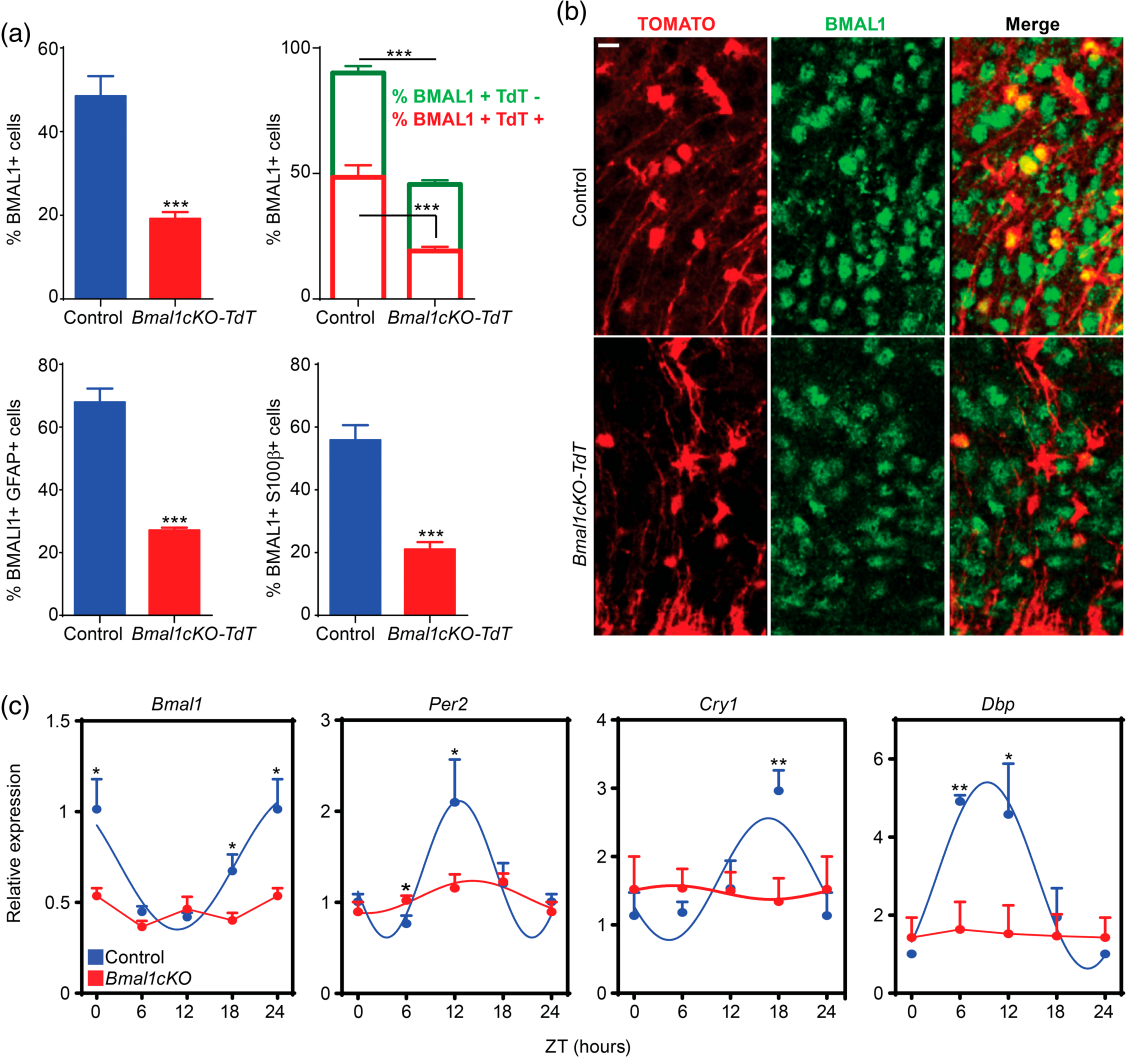
BMAL1 deletion in astrocytes globally impairs the hypothalamic molecular clock. (a) Upper left panel, a reduction of BMAL1 positive cells was observed in the ARC nucleus of *Bmal1cKO* mice compared with control animals 2 months after TM treatment (*Y*‐axis represents the percentage of total BMAL1‐positive cells in the ARC nucleus). Data are represented as mean ± SEM (*n* = 4, paired *t*‐test, ***p < .001 vs. control animals). Upper right panel, a 60% reduction of BMAL1‐positive cells was observed in the population of Td‐TOMATO‐positive cells of *Bmal1cKO* compared with control animals (red, paired *t*‐test, ***p < .001 vs. control animals). A 36% reduction of BMAL1‐positive cells in the population of Td‐TOMATO‐negative cells was found in *Bmal1cKO* compared with control animals (green, paired *t*‐test ***p < .001 vs. control animals). Data are represented as mean ± SEM (*n* = 4). Percent of BMAL1‐positive cells was significantly reduced in GFAP (lower left panel) or S100β (lower right panel) positive astrocytes in the ARC nucleus of *Bmal1cKO*‐*Td‐Tomato* mice compared with control animals. Data are represented as mean ± SEM (*n* = 4, paired *t*‐test, ***p < .001 vs. control animals). (b) Representative micrographs of BMAL1 immunostaining in the ARC nucleus of control or *Bmal1*cKO‐*Td‐Tomato* animals. Scale bar, 25 μm. (c) Analysis of clock transcripts (*Bmal1*, *Cry1*, *Per2* and BMAL1 target, *Dbp*) in the hypothalamus of control and *Bmal1cKO* mice after 2 months of TM treatment. Experimental data were cosine fitted. The ZT24 time point is the ZT0 time point, shown again. Data are represented as mean ± SEM (*n* = 5–6, paired *t*‐test, *p < .05 and **p < .01) [Color figure can be viewed at wileyonlinelibrary.com]

To test our hypothesis, we crossed *Bmal1cKO* mice with a Cre‐inducible Red fluorescent reporter mouse line (*Td‐Tomato*), as we previously reported (Barca‐Mayo et al., 2017). We first ascertained the specificity and efficiency of GLAST‐Cre‐mediated BMAL1 deletion in hypothalamic astrocytes, by quantifying the co‐immunolocalization of TOMATO with the astrocyte markers Glial fibrillary acidic protein (GFAP) or S100 calcium‐binding protein β (S100β) and BMAL1 in *Glast‐Cre‐Td‐Tomato* (control) or *Bmal1cKO‐Td‐Tomato* animals. As expected, 2 months after TM, virtually all of the Cre‐recombined cells in the hypothalamus, as revealed by TOMATO, exhibited the stellated morphology characteristic of astrocytes, as well as immunoreactivity for GFAP and S100β (Table S2), consistently with previous observations (García‐Cáceres et al., 2016). BMAL1 was expressed in 48.5 ± 4.78% of TOMATO positive astrocytes in the ARC nucleus of control animals. In contrast, this proportion was reduced by approximately 60% in *Bmal1cKO‐Td‐Tomato* mice (paired *t* test, *p* = 8.39 × 10^−6^; Figure 3a,b). Similarly, the percentage of GFAP or S100β positive astrocytes expressing BMAL1 was significantly reduced by 60.1 and 62.3%, respectively, in the ARC of *Bmal1cKO‐Td‐Tomato* mice (paired *t* test, *p* = 6.9 × 10^−8^ for GFAP and *p* = 8.88 × 10^−5^ for S100β), compared to controls (Figure 3a).

Remarkably, in the mutants, the percentage of BMAL1 positive cells was also reduced by 36% in TOMATO negative cells (paired *t* test, *p* = 9.27 × 10^−5^; Figure 3a,b), suggesting that deletion of BMAL1 in GLAST positive astrocytes might also impact the clock in other cell populations of the hypothalamus, as we previously reported in cortex and hippocampus (Barca‐Mayo et al., 2017). To evaluate the impact of BMAL11 depletion on global oscillations in the hypothalamus, we quantified rhythmic expression of clock genes in controls and *Bmal1cKOs*, 2 months after TM treatment, at different ZTs. In control mice, we found rhythmic expression of *Bmal1*, *Cry1*, *Per2*, and BMAL1‐target *Dbp*, as previously reported (Barca‐Mayo et al., 2017) (Figure 3c). However, these oscillations were attenuated in *Bmal1cKOs* (Figure 3c), indicating that the deletion of BMAL1 in astrocytes globally impairs the molecular clock and, therefore, the circadian function of the hypothalamus.

### 3.4 *Bmal1cKO* mice showed age‐dependent astrogliosis and apoptosis of hypothalamic astrocytes

**Figure 4:**
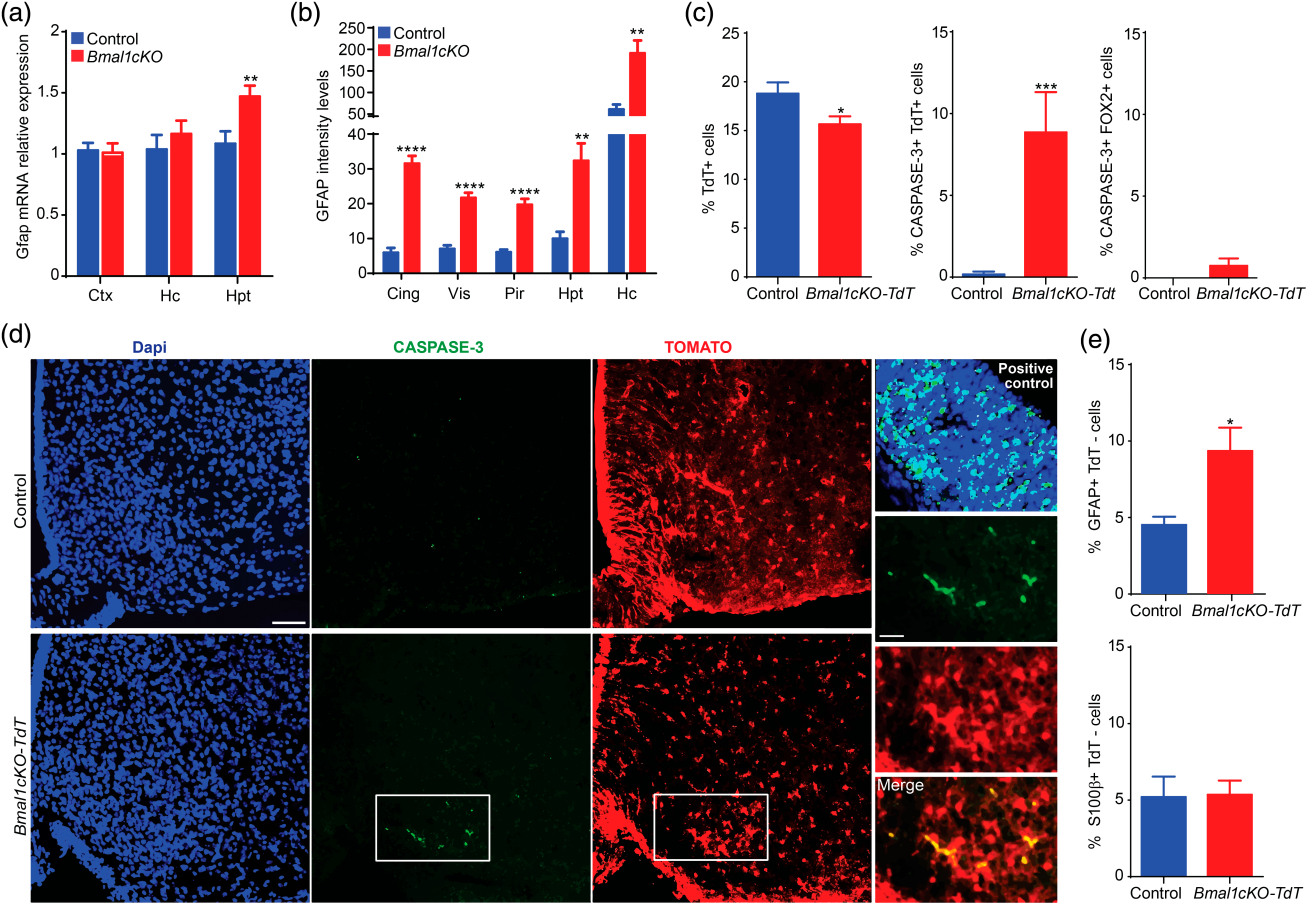
*Bmal1cKO* mice showed age‐dependent astrogliosis and apoptosis of hypothalamic astrocytes. (a) *Gfap* expression in cortex (Ctx), hippocampus (Hc) and hypothalamus (Hpt) of control and *Bmal1cKO* mice, 2 months after TM treatment. Data are represented as mean ± SEM (*n* = 5, paired *t*‐test, **p < .01). (b) Quantification of GFAP fluorescence intensity in cingulate (Cing), visual (Vis), piriform (Pir) cortex, Hpt and Hc of *Bmal1cKOs*, and controls, 4 months after TM treatment. Data are represented as mean ± SEM (*n* = 5, paired *t*‐test, **p < .01 and ****p < .0001 vs. controls). (c) Left panel, percentage of TOMATO‐positive cells in the ARC nucleus of control (*Glast‐Cre‐Td‐Tomato*) or *Bmal1cKO‐Td‐Tomato* animals, 2 months after TM treatment. Middle and right panel, percentage of active‐CASPASE 3 cells that co‐localized with TOMATO or FOX2 positive cells, respectively. Data are represented as mean ± SEM (*n* = 5, paired *t*‐test, *p < .05 and ***p < .001 vs. controls). (d) Representative micrographs of TOMATO and active CASPASE‐3 immunostaining in the ARC nucleus of control and *Bmal1cKO‐Td‐Tomato* mice 2 months after TM treatment. Scale bars, 50 and 25 μm in the higher magnification images. Cortex of *Dgrc8* (DiGeorge Syndrome Critical Region Gene) knockout mice at embryonic day 13.5 was used as a positive control (right upper panel). (e) Percentage of GFAP (upper panel) or S100β (lower panel) positive cells that are TOMATO‐negative in the ARC nucleus of control and *Bmal1cKO‐Td‐Tomato* animals. Data are represented as mean ± SEM (*n* = 5, paired *t*‐test, *p < .05 vs. controls) [Color figure can be viewed at wileyonlinelibrary.com]

Next, we investigated whether the deregulation of the molecular clock in the brains of our mutants lead to reactive gliosis, as previously reported in cortex and hippocampus of constitutive *Bmal1*−/−, *Nestin‐Cre‐Bmal1* or *Aldh1l1‐CreERT2‐Bmal1* mice (Lananna et al., 2018; Musiek et al., 2013; Nakazato et al., 2017). Increased GFAP levels are generally regarded as hallmark of reactive glia, therefore we quantified *Gfap* transcripts or protein by qPCR or immunostaining, respectively, in different brain areas (cortical, hippocampal, and hypothalamic regions) of control and *Bmal1cKO* mice at 2 and 4 months after TM treatment. In agreement with previous reports (Lananna et al., 2018; Musiek et al., 2013; Nakazato et al., 2017), we did not detect the increased expression of *Gfap* in cortex or hippocampus 2 months after TM treatment (Figure 4a, Table S1). However, 4 months after TM treatment, we found increased GFAP immunoreactivity in different cortical regions and hippocampus of the mutants (Figure 4b and S1), confirming that *Bmal1cKO*s developed age‐dependent gliosis. Surprisingly, already at 2 months after TM treatment, we found elevated expression of *Gfap* in the hypothalamus of *Bmal1cKO* compared to control mice (Figure 4a). This result suggests that BMAL1 deletion in astrocytes leads to a temporal activation of this glial cell type which differs among different brain areas and, importantly that the hypothalamus is, among the different brain regions analyzed, the first in showing reactive gliosis.

To gain further insights in this observation, we investigated whether reactivity was restricted to the subpopulation of GLAST positive astrocytes, in two different areas of the hypothalamus: The ARC nucleus, involved in the control of food intake and where inflammation and gliosis was linked to obesity in rodents and humans (reviewed in Valdearcos et al., 2015; McNay et al., 2012; Thaler et al., 2012) and the SCN, the main regulator of circadian locomotor activity in mammals (Ralph, Foster, Davis, & Menaker, 1990; Stephan & Zucker, 1972). Surprisingly, we found a 17% reduction of GLAST positive astrocytes (as revealed by TOMATO) in the ARC of *Bmal1cKO‐Td‐Tomato* mice compared to control animals, 2 months after TM treatment (Figure 4c). Moreover, we detected a significant decrease in the total number of cells per area, as determined by DAPI staining, in the ARC of the mutants 2 months after TM treatment (469.44 ± 39.99 for *Bmal1cKOs* vs. 585.14 ± 38.05 for controls, paired *t* test, *p* = .046). This decrease in the total cell numbers in the ARC of *Bmal1cKO* mice was not due to a reduction in the number of neurons, as shown by quantification of the neuronal marker RNA Binding Fox‐1 Homolog 2 (FOX2), a homolog of NeuN (Kim, Adelstein, & Kawamoto, 2009; Underwood, Boutz, Dougherty, Stoilov, & Black, 2005) (50.11% + 1.76 for controls and 53.49% + 2.64 for *Bmal1cKOs*, paired *t*‐test, *p* = .29). Indeed, in the ARC nucleus of *Bmal1cKO‐Td‐Tomato* mice we found a significant increase in the proportion of TOMATO‐positive astrocytes, but not in FOX2 positive neurons, that were also positive for the apoptosis marker active‐CASPASE‐3 (Figure 4c,d). Additionally, we observed a significant increase in the proportion of TOMATO negative cells that were positive for GFAP, while no differences were found in the expression of S100β among TOMATO positive or negative cells (Figure 4e and S2). Altogether, these results indicate a selective loss of GLAST positive astrocytes upon deletion of BMAL1 (i.e., TOMATO positive cells), and reactivity in the astrocytes that retained BMAL1 expression (i.e., TOMATO‐negative cells).

As we found reduced astrocyte numbers and increased the reactivity of astrocytes in the ARC after 2 months of TM treatment, we evaluated whether *Bmal1* deletion was maintained at later time, specifically at 15 months post tamoxifen treatment. We found that the percent of GFAP positive cells was increased in the ARC nucleus of both control and *Bmal1cKO* mice with age (Figure S3b) as previously reported (reviewed in Palmer & Ousman, 2018). However, at this stage, *Bmal1cKOs* showed a significant increase in the percent of GFAP positive cells compared to control animals (Figure S3a,b). On the other hand, the percentage of BMAL1 positive cells in the ARC of control animals was reduced at 15 months after TM treatment compared to 2 months posttreatment (Figure S3c). This observation is consistent with previous reports showing a decline in the clock gene expression with age (reviewed in Hood & Amir, 2017). Despite, at this stage, we observed a reduction in the percentage of total BMAL1 positive cells in the ARC of the mutants compared to control animals, it did not reach significance (two‐way ANOVA, *p* = .19; Figure S3a,c). However, co‐immunostaining of GFAP and BMAL1 indicated a significant reduction among the BMAL1 positive that were GFAP positive or negative in the mutants (Figure S3d). Therefore, these results suggest that BMAL1 deletion is maintained at later time points after TM treatment. Indeed, despite that we detected a significant increase in the percentage of GFAP‐positive cells that were mitotically active (by coimmunostaining with the proliferative marker, KI67) after 15 months of TM treatment in the ARC nucleus of the mutants as compared to control animals (0.5% vs. 0.17%, respectively), this percentages were very small (Figure S4) and likely biologically irrelevant. Consistent with a previous report in *Bmal1nestin−/−* mice, these data suggest that the deletion of *Bmal1* does not increase the number of astrocytes but rather increases the activation of pre‐existing ones (Nakazato et al., 2017).

Remarkably, in the SCN, no differences between control and *Bmal1cKO* animals was observed in the percentage of TOMATO (17.84% ± 1.51 vs. 17.23% ± 1.12, respectively, paired *t* test, *p* = .74), GFAP (16.47% ± 0.92 vs. 19.15% ± 0.82, respectively, paired *t* test, *p* = .58) or FOX2 positive cells (8.38% ± 0.46 vs. 10.5% ± 2, respectively, paired *t* test, *p* = .63), 2 months after TM treatment. Consistently, no differences were found in the proportion of TOMATO negative cells that were positive for GFAP between control and *Bmal1cKO* animals (7.11% ± 0.94 vs. 7.10% ± 0.72, respectively, paired *t*‐test, *p* = .99) indicating that BMAL1 deletion in SCN astrocytes do not lead to reactive gliosis 2 months after TM. As wheel‐running activity is widely used as an index of SCN circadian function (Ralph et al., 1990; Stephan & Zucker, 1972), this observation is consistent with the comparable pattern of daily activity in control and mutant animals in 12 hr–12 hr light–dark cycles (Figure 2b) indicating that the SCN circadian function was not altered in *Bmal1cKO* mice 2 months after TM treatment. It was reported that the circadian output measured at the level of circadian locomotor behavior is dampened with age (Nakamura et al., 2011). Consistent with this report, we found that the daily activity of control animals, at 15 months after TM treatment, were reduced as compared to younger mice (2 months after TM treatment; Figures 2b and S5). Remarkably, the circadian locomotor activity of the mutants, at this stage, was reduced as compared to controls (Figure S5).

Our results indicate that BMAL1 deletion in astrocytes lead to a temporal‐ and regional‐specific gliosis within the hypothalamus and, importantly that ARC nucleus is, among the different brain regions analyzed, the first showing astrocyte reactivity and apoptosis. This cascade of events likely accounts for the altered hypothalamic function of *Bmal1cKO* mice, which might lead to the increased body weight and metabolic alterations of the mutants. Together, these results (i.e., shorter lifespan, glucose imbalance, altered growth curves, age‐dependent gliosis and decrease of age‐related circadian locomotor activity) support our hypothesis that BMAL1 deletion in astrocytes may lead to premature aging.

### 3.5 GABAA receptor antagonist delayed the aging and metabolic phenotype of *Bmal1cKO* mice

**Figure 5:**
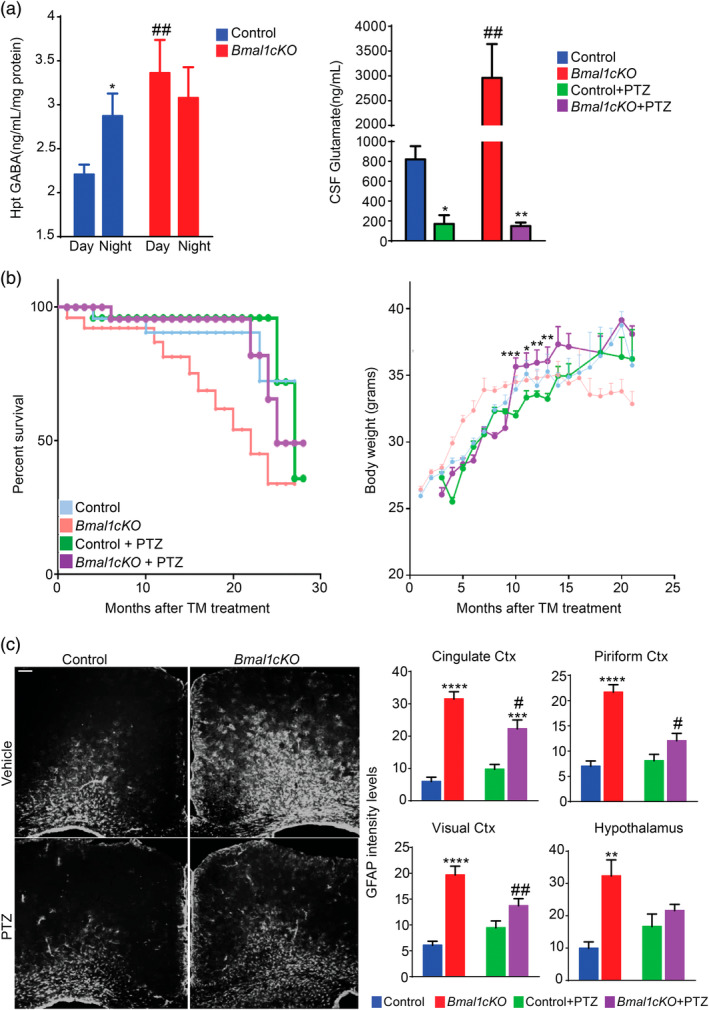
GABAA receptor antagonists delayed the aging and metabolic phenotype of *Bmal1cKO* mice. (a) Left panel, GABA levels in the hypothalamus of *Bmal1cKOs* and controls at day and night time, 2 months after TM treatment. Data are represented as mean ± SEM (*n* = 5, two‐way ANOVA, *p < .05 vs. daytime and ##*p* < .01 vs. controls). Right panel, CSF glutamate levels of naive or PTZ‐treated *Bmal1cKO* and control animals at ZT6. Data are represented as mean ± SEM (*n* = 5, two‐way ANOVA, *p < .05 and **p < .01 vs. naive animals; ##*p* < .01 vs. controls). (b) Left panel, Kaplan–Meyer survival curve of naive or PTZ‐treated control and *Bmal1cKO* mice. Right panel, age‐dependent changes in body weight of control and *Bmal1cKO* mice treated with PTZ. Data are represented as mean ± SEM (paired *t*‐test, *p < .05, **p < .01, and ***p < .001 vs. PTZ‐treated control animals). *n*  = 10– 12 for PTZ‐treated control and *Bmal1cKO* animals. (c) Left panel, representative micrographs of GFAP immunostaining in the cingulate cortex of naive or PTZ‐treated control and *Bmal1cKO* mice, 4 months after TM treatment. Scale bar, 100 μm. Right panel, quantification of fluorescence intensity of GFAP levels in cingulate, piriform, and visual cortex (Ctx) as well as in the hypothalamus of naive or PTZ‐treated *Bmal1cKO* and control animals. Data are represented as mean ± SEM (*n* = 5, two‐way ANOVA, **p < .01, ***p < .001 and ****p < .0001 vs. control animals and #*p* < .05 and ##*p* < .01 vs. naive mice) [Color figure can be viewed at wileyonlinelibrary.com]

Hypothalamic neurons expressing the orexigenic (appetite‐increasing) Neuropeptide Y/Agouti‐related peptide (NPY/AGRP) or the anorexigenic (appetite‐suppressing) Pro‐opiomelanocortin (POMC) receive inhibitory and excitatory inputs from GABA and glutamate. These two neurotransmitters account for most of the synaptic activity in the hypothalamus and therefore, are directly involved in appetite and energy balance regulation (reviewed in Delgado, 2013). We recently reported increased GABA levels in the CSF of *Bmal1cKO* mice in the light phase (day time, i.e., ZT6), 2 months after TM treatment (Barca‐Mayo et al., 2017). We now expand on these results by showing significantly higher levels of GABA also in the hypothalamus of *Bmal1cKO* mice in the light phase, compared to control animals (Figure 5a). Therefore, we hypothesized that the increased levels of GABA in the CSF and hypothalamus of *Bmal1cKOs* might be associated to their metabolic and age‐associated dysfunctions. Consistently, it was reported a stimulatory role for GABA in the regulation of hypothalamus‐dependent feeding behavior. Specifically, intracerebroventricular (ICV) administration of the GABAA receptor agonist muscimol stimulates feeding in satiated pigs, a response that is prevented by the specific GABAA receptor antagonist bicuculline (Baldwin, Ebenezer, & De La Riva, 1990). Also, systemic and ICV administration of the GABAB receptor agonist baclofen increases food intake in satiated pigs that can be abolished by pretreatment with the GABAB receptor antagonist phaclofen (Ebenezer & Baldwin, 1990).

On the other hand, glutamate stimulates feeding upon the administration of its receptor agonists. ICV or lateral hypothalamic injection of glutamate, or its excitatory amino acid agonists (kainic acid, d,l‐alpha‐amino‐3‐hydroxy‐5‐methyl‐isoxazole propionic acid, and N‐methyl‐d‐aspartic acid) rapidly elicits intense food intake in rats (Stanley, Ha, Spears, & Dee, 1993; Stricker‐Krongrad, Beck, Nicolas, & Burlet, 1992). Likewise, ICV administration of metabotropic glutamate receptor 5 (mGluR5) agonists stimulate feeding whereas the mGluR5 receptor antagonist (R,S)‐2‐chloro‐5‐hydroxyphenylglycine, inhibits food intake (Ploj et al., 2010). Thereby, we also quantified glutamate levels in the CSF of control and *Bmal1cKO* animals 2 months after TM treatment at ZT6, following a previously described liquid chromatography–tandem mass spectrometry protocol (Buck, Voehringer, & Ferger, 2009). Control animals showed CSF glutamate levels in line with those previously reported (~7 μg ml^−1^; Espey, Kustova, Sei, & Basile, 1998). Remarkably, glutamate levels were significantly higher in the CSF of *Bmal1cKOs* (Figure 5a).

Previously, we showed that the increased GABA levels in the CSF of *Bmal1cKO* leads to the over‐inhibition of the circuits involved in learning and memory and to the uncoupling the SCN oscillators (Barca‐Mayo et al., 2017). Consistently, administration of GABAA receptor antagonists, following previously reported protocols (Colas et al., 2013; Fernandez et al., 2014; Ruby et al., 2013), restored the circadian locomotor activity and the cognitive functions of the mutants (Barca‐Mayo et al., 2017). To our surprise, we now found that glutamate levels in the CSF of both control and *Bmal1cKO* mice were strongly downregulated below normal levels 1 week after administration of the GABAA receptor antagonist pentylenetetrazole (PTZ, administered 2 months after TM treatment; Figure 5a). Importantly, the daily administration of PTZ at ZT6 (for 10 consecutive days during the pre‐obese stage) was sufficient to delay the transient obesity of *Bmal1cKO* mice for 6 months, while no differences were found in control animals (Figure 5b). Strikingly, PTZ treatment normalized the lifespan of *Bmal1cKO* animals (average lifespan 22 vs. 25 months after TM treatment, respectively, Log‐rank test, *p* = .049) while no differences were found in controls (Figure 5b). The long‐term effect of this protocol of PTZ treatment, at nonepileptic doses, is not surprising at it was previously shown to produce long‐lasting cognitive improvements after drug withdrawal in rodents (Colas et al., 2013; Contestabile, Magara, & Cancedda, 2017; Fernandez et al., 2014; Ruby et al., 2013). Interestingly, we also found that PTZ‐treated *Bmal1cKO* mice displayed significantly reduced levels of reactive gliosis in different areas of the cortex as well as in the hypothalamus in comparison to untreated animals at 4 months after TM treatment (i.e., 2 months after the PTZ administration; Figure 5c).

In sum, as the administration of GABAA receptor antagonist normalized glutamate levels, reduced astrocytes reactivity, delayed obesity and increased the lifespan of *Bmal1cKO* mice, we postulate that GABA and/or glutamate‐signaling are likely involved in astrocyte‐dependent control of lifespan and energy balance.

## 4 DISCUSSION

This study is the first demonstration that adult deletion of BMAL1 in a subpopulation of astrocytes is sufficient to shorten lifespan and alter energy balance, thus partly recapitulating phenotypes described in constitutive *Bmal1−/−* (Bunger et al., 2005; Kondratov et al., 2006; Lamia et al., 2008; Lee et al., 2006; Marcheva et al., 2010; Rudic et al., 2004; Shi et al., 2006). Importantly, as modulation of GABAA‐receptor signaling delayed the metabolic dysfunctions and the early death of *Bmal1cKO* mice, we demonstrate a crucial contribution of astrocytic clock in linking GABA signaling with the circadian regulation of metabolism and lifespan.

Astrocytes are widely distributed throughout the nervous system and express brain‐region‐specific genes. Thereby the selection of regulatory elements to target all astrocytes in vivo is almost impossible with today's tools. However, the general practice when using genetic approaches to perform recombination in all (or most) target cells might not be needed for astrocytes‐mediated phenotypes due to their anatomical properties. First, astrocytes are organized in structurally nonoverlapping domains in vivo where one astrocyte interacts with four neuron cell bodies, between 300 and 600 dendrites and more than 100,000 synapses (Bushong, Martone, Jones, & Ellisman, 2002; Halassa, Fellin, & Haydon, 2007). Second, astrocytes are interconnected through gap junction channels which allow metabolic or biochemical coupling with propagation distances ranging from four to up to 30 astrocytes (reviewed in Tian et al., 2006; Sul, Orosz, Givens, & Haydon, 2004). Therefore, despite not all astrocytes are directly targeted in our *Bmal1cKO* mice, the finding that they partially recapitulate the metabolic phenotype and early death of constitutive *Bmal1*−/− mice is not surprising and supported by the recent report that few GFAP positive astrocytes of the SCN are sufficient to instruct neurons to initiate and indefinitely sustain circadian patterns of activity and behavior (Brancaccio et al., 2019).

Our findings that the administration of a GABAA receptor antagonist normalized the glutamate levels, delayed the reactive gliosis and the metabolic phenotypes and increased the lifespan of *Bmal1cKO* mice, suggest that the altered GABA and/or glutamate astrocyte–neuron coupling underlie the phenotypes of our mutants. This hypothesis is supported by previous reports showing that astrocytes critically modulate hypothalamic neural circuits controlling energy homeostasis (Chen et al., 2016; Zhang, Reichel, Han, Zuniga‐Hertz, & Cai, 2017) by modulating extracellular GABA bioavailability (Zhang et al., 2017). Additionally, astrocytes can impact the molecular clock in cortical, hippocampal, SCN (Barca‐Mayo et al., 2017; Barca‐Mayo, Berdondini, & De Pietri Tonelli, 2019; Tso et al., 2017; Duhart et al., 2013) and hypothalamic neurons (present study). Indeed, we and others previously reported that GABA uptake and glutamate release coupled to astrocyte rhythms (Barca‐Mayo et al., 2017; Brancaccio et al., 2017, 2019; Tso et al., 2017) play a key role for rhythmic astrocyte‐neuron intercellular communication. In line with these observations, our study opens the possibility that astrocyte rhythmic regulation of GABA and/or glutamate might transmit timing information between this glial cell type and hypothalamic neuronal networks to optimize energy balance.

An important implication of our study is that while the deletion of BMAL1 is local, the impact on circulating leptin, glucose, and insulin provides a new link among circadian timing in astrocytes and systemic metabolic states or peripheral clocks. In the first case, by expressing the receptors for leptin and insulin (Cheunsuang & Morris, 2005; García‐Cáceres et al., 2016; Hsuchou et al., 2010; Hsuchou, Pan, Barnes, & Kastin, 2009; Jayaram et al., 2013; Kim et al., 2014; Pan et al., 2008), astrocytes are endowed with metabolic signal‐sensing properties, suggesting an alternative role for the regulation of energy homeostasis. Indeed, astrocytes have been reported to sense insulin and leptin, as well as to co‐regulate behavioral responses and metabolic processes via the control of brain glucose uptake and the glial ensheathment of POMC neurons in the ARC, respectively (García‐Cáceres et al., 2016; Kim et al., 2014), However, it is also conceivable that astrocyte clock control of leptin and glucose homeostasis may occur through interactions with peripheral clocks, leading to altered leptin rhythms or to inadequate response of peripheral tissues to circulating insulin respectively in the mutants. For example, astrocytes, as well‐known targets of glucocorticoids, might be sensitive to the negative feedback loop of the hypothalamo‐pituitary‐adrenal axis, thus linking peripheral and central oscillators. Importantly, it is widely accepted that while glucocorticoid signaling can reset peripheral clocks, it does not impact the central clock because SCN neurons do not express the glucocorticoid receptor Nuclear Receptor Subfamily 3 Group C Member 1 (NR3C1; Rosenfeld, Van Eekelen, Levine, & De Kloet, 1988). Thus, astrocytic feedback loops, via glucocorticoid signaling, could explain the so far puzzling results showing that the Per1‐Luc phases of SCN were affected significantly when adrenalectomized animals were treated with hydrocortisone administered in their drinking water (Pezük, Mohawk, Wang, & Menaker, 2012). It will be an enticing challenge to identify astrocyte rhythmic outputs or inputs from systemic cues, which transmits timing information between this glial cell type and peripheral tissues and/or clocks to optimize energy balance.

The effect of astrocytic BMAL1 deletion on the metabolic dysfunctions and early death cannot be completely disentangled from the effects on SCN‐mediated rhythms. However, several evidences suggest that these phenotypes of *Bmal1cKO* mice rely in extra‐SCN clocks. First, the circadian locomotor activity, commonly used as an index of SCN circadian function (Ralph et al., 1990; Stephan & Zucker, 1972), is not lost in mice with *Bmal1* deletion in SCN astrocytes (Barca‐Mayo et al., 2017; Brancaccio et al., 2017; Tso et al., 2017). Second, BMAL1 deletion in SCN does not affect either lifespan or body weight despite complete loss of rhythmic behavior (Izumo et al., 2014). Consistently the circadian locomotor activity but not the ageing and metabolic disturbances of *Bmal1−/−* mice was rescued by restoring BMAL1 expression in the SCN (McDearmon et al., 2006). Third, BMAL1 specific ablation within steroidogenic factor 1 (SF1)‐neurons of the ventromedial hypothalamus is sufficient to alter energy expenditure (Orozco‐Solis et al., 2016). This study is in contrast with a report showing no alterations body weight and glucose homeostasis upon adult BMAL1 deletion, driven by tamoxifen‐regulated activation of the estrogen receptor 1 (*Esr1*)‐Cre system (Yang et al., 2016) selectively targeting SF1‐positive neurons, which express ESR1 (Musatov et al., 2007). A possible explanation for this apparent contradiction is that the timing of BMAL1 deletion, postnatally or from development, respectively, influences its effects on aging and survival (Yang et al., 2016). Interestingly, at present, expression of ESR1 has not been reported in hypothalamic astrocytes in vivo (Liu & Shi, 2015) thus providing a possible explanation for the phenotypic differences between *Glast‐cre* and *Esr1‐cre‐driven Bma1cKO* mice. Altogether, this suggests that a functional molecular clock in SCN astrocytes is neither sufficient nor required for controlling lifespan and energy balance.

Transcriptional activity induced by BMAL1 may have effects independent of the circadian clock that could impinge on aging‐associated dysfunctions and metabolism. Indeed, distinguishing the specific importance of circadian oscillation versus the “static” function of clock genes is a major challenge for all studies involving genetic manipulations of core clock genes. However, the BMAL1 DNA binding is highly rhythmic and regulated by the clock (Koike et al., 2012), making it difficult to separate entirely from circadian rhythms. For example, the reactive gliosis and astrocyte dysfunction observed in a neuronal‐specific *Bmal1KO* (Izumo et al., 2014) or in mice upon circadian disruption by exposure to 10 hr:10 hr light–dark conditions, which blunts BMAL1 oscillations in the brain (Lananna et al., 2018), suggest that it might be due to the loss of rhythms. On the other hand, it was also reported that arrhythmicity in the setting of increased BMAL1 expression, as in *Per1/2* mutant mice, does not induce astrocyte activation (Lananna et al., 2018). Therefore, it is difficult to discern whether the reactive gliosis is dependent on suppression of BMAL1‐mediated transcription or to the loss of rhythms.

Reactivity in astrocytes typically increases their inflammatory phenotype and cause loss of their neuro‐supportive functions, thus rendering neurons vulnerable to hypo‐metabolic states, excitotoxicity and oxidative stress. Thus, gliosis is considered general hallmark of brain aging (Musiek et al., 2013) and age associated neurodegenerative disorders (reviewed in Camandola & Mattson, 2017). Importantly, reactive astrocytes abnormally produce and release GABA (Jo et al., 2014) and decrease glutamate uptake (Beurrier et al., 2010; Escartin et al., 2006). Indeed, suppressing GABA production or release from reactive astrocytes fully restores the synaptic plasticity and memory in a mouse model with Alzheimer's disease (Jo et al., 2014). On the other hand, aging is associated with increased levels of reactive oxygen species (ROS) and oxidized products in different tissues (reviewed in Balaban, Nemoto, & Finkel, 2005). Indeed, the free radical theory of aging postulates that the production of intracellular ROS is the major determinant of lifespan (reviewed in Balaban et al. (2005)). Remarkably, the cellular redox state is dependent on BMAL1 expression (Khapre, Kondratova, Susova, & Kondratov, 2011; Wang et al., 2012) and the acetylation of multiple critical mitochondrial proteins shows circadian oscillation, indicating clock‐mediated control of the redox state (Masri et al., 2013). ROS are important regulators of cellular metabolism, gene expression, and other molecular responses, playing key roles in the control of various physiological processes. The levels of external (food‐generated) and internal (metabolism/activity‐generated) oxidants change during the day as a result of fluctuations in food intake and behavior. Therefore, control of ROS homeostasis by the circadian system, which is intrinsically connected to an organism's daily activity, would provide the most effective protection from the damaging effects of oxidants at any given time of day. Indeed, the early aging phenotype in *Bmal1*−/− animals correlates with increased levels of ROS in some tissues, including brain, and treatment with the glutathione precursor N‐acetyl cysteine, extends lifespan in these mice (Kondratov et al., 2006; Kondratov, Vykhovanets, Kondratova, & Antoch, 2009; Kondratova, Dubrovsky, Antoch, & Kondratov, 2010). Similarly, targeted deletion of BMAL1 in neurons and glia promoted neuronal death in primary cultures and in mice treated with a chemical inducer of oxidative injury and neurodegeneration (Musiek et al., 2013). Consistent with our results, it was previously shown that BMAL1 deletion in astrocytes induces gliosis and inflammatory gene expression in vitro and in vivo, mediated in part by suppression of glutathione‐S‐transferase signaling (Lananna et al., 2018). Remarkably, supplementing BMAL1‐deficient astrocyte cultures or mice with NAC prevents astrogliosis (Lananna et al., 2018). Our results showing a lost in hypothalamic astrocytes upon BMAL1 deletion as well as the finding that the hypothalamus is the first region of the brain showing the reactive gliosis, suggest that arrhythmic astrocytes might lead to an inflammatory and hypometabolic state, rendering neurons to be more susceptible for neurodegeneration, excitotoxicity, and oxidative stress. Indeed, loss of BMAL1 in astrocytes promotes neuronal death in vitro to influence many aspects of brain function and neurological disease (Musiek et al., 2013). Failure of neurons to respond adaptively to a decline in basal metabolic rate and in energy‐driven tasks is a risk factor for age associated neurodegenerative disorders. At the same time, an imbalance between the circadian and ROS generating/metabolizing systems might increase damage due to oxidative stress, thus contributing to and/or complicating pathogenesis, aging, and lifespan.

Our finding that BMAL1 loss in astrocytes leads to increased GABA and glutamate levels and to age‐dependent gliosis has many potential implications for age‐related neurodegenerative diseases and suggests that further study of the regulation and function of astrocyte core clock genes in health and disease is warranted. Therefore, here we propose that circadian manipulation of GABA signaling, GABA uptake by astrocytes and/or bolstering astrocyte clock might have neuroprotective effects in noninvasive therapies for metabolic disorders and ageing.

## CONFLICT OF INTEREST

The Authors declare that there is no conflict of interest.

## Supporting information


**Appendix S1**: Supplementary material

## Data Availability

All raw/original relevant data are available upon request.
