# Supplementary material for: Deletion of astrocytic BMAL1 results in metabolic imbalance and shorter lifespan in mice
Source: Glia. 2019 Dec 13;68(6):1131–47. doi: 10.1002/glia.23764 (PMC7496695; doi:10.1002/glia.23764)
Supplement: Supplementary file 1 — Appendix S1: Supplementary material [file GLIA-68-1131-s001.docx]

## Supplementary material for the manuscript

**Full title:** Deletion of astrocytic BMAL1 results in metabolic imbalance and shorter lifespan in mice

**Authors:** Olga Barca-Mayo^1*^, Arjen J. Boender^2^, Andrea Armirotti^3^ and Davide De Pietri Tonelli^1^

^1^Neurobiology of miRNA lab. Fondazione Istituto Italiano di Tecnologia, Genoa, 16163, Italy.

^2^Neuromodulation of Cortical and Subcortical Circuits Lab. Fondazione Istituto Italiano di Tecnologia, Genoa, 16163, Italy.

^3^D3 PharmaChemistry, Fondazione Istituto Italiano di Tecnologia, Genoa, 16163, Italy.

*Address correspondence to: Olga Barca-Mayo, Circadian and Glial Biology Lab, Molecular Medicine and Chronic Diseases Research Centre (CiMUS), University of Santiago de Compostela, Santiago de Compostela, 15706, Spain. Email: olga.barca.mayo@usc.es Phone: +34 604077749.

**Content**

**Table S1** Comparison of the phenotypes of *Bma1cKO* and constitutive *Bmal1* -/- mice.

**Table S2** Specificity and efficiency of inducible GLAST-Cre-mediated recombination.

**Figure S1** Representative micrographs of GFAP immunostaining in control and *Bmal1cKO* mice.

**Figure S2** GFAP and TOMATO co-localization in the ARC nucleus of control and *Bmal1cKO* animals.

**Figure S3** BMAL1 deletion in ARC astrocytes at 15 months after TM treatment.

**Figure S4** Quantification of KI67 positive astrocytes in the ARC of control and mutants at 15 months after TM treatment.

**Figure S5** Daily activity of control and *Bmal1cKO* mice 15 months after TM treatment.

|  | ***Bmal1cKO (time after TM treatment)*** | **Constitutive *Bmal1* -/-** |
| --- | --- | --- |
| **Average Lifespan** | 22 months | 6-9 months  (Bunger et al., 2005; Lee et al., 2006; Kondratov et al., 2006) |
| **Age-dependent astroglial activation** | Hypothalamus (ARC): From 2 months  Cortex, Hippocampus: From 4 months | Cortex, Hippocampus: from 2.5 months (Musiek et al., 2013; Lanana et al., 2018) |
| **Increased body weight** | From 4-8 months | From 1-2 months of age  (Lamia, et al., 2008) |
| **Decreased body weight** | From 19 months | From 4-5 months  (Bunger et al., 2005; Kondratov et al., 2006; Lee et al., 2006) |
| **Increased Insulin sensitivity** | From 3 months | 2-3 months  (Rudic et al., 2004; Lamia, et al., 2008) |
| **Insulin resistance** | From 6 months | From 2-3months  (Shi et al., 2006; Lamia, et al., 2008) |

**Table S1** Comparison of the phenotypes of *Bma1cKO* mice and constitutive *Bmal1* -/- mice

Bunger, M. K., Walisser, J. A., Sullivan, R., Manley, P. A., Moran, S. M., Kalscheur, V. L., … & Bradfield, C. A. (2005). Progressive arthropathy in mice with a targeted disruption of the Mop3/Bmal-1 locus. *Genesis*, **41**( 3), 122- 32. https://doi.org/10.1002/gene.20102

Lee, S., Donehower, L. A., Herron, A. J., Moore, D. D., & Fu, L. (2006). Disrupting circadian homeostasis of sympathetic signaling promotes tumor development in mice. *PloS one*, **5**( 6), e10995. [https://doi.org/10.1371/journal.pone.0010995](%20https://doi.org/10.1371/journal.pone.0010995)

Kondratov, R. V., Kondratova A. A., Gorbacheva, V. Y., Vykhovanets, O. V., & Antoch, M. P. (2006). Early aging and age-related pathologies in mice deficient in BMAL1, the core component of the circadian clock. *Genes Development*, **15**( 14), 1868- 73. <https://doi.org/10.1101/gad.1432206>

Musiek, E. S., Lim, M. M., Yang, G., Bauer, A. Q., Qi, L., Lee, Y., … Fitzgerald, G. A. (2013). Circadian clock proteins regulate neuronal redox homeostasis and neurodegeneration. *Journal of Clinical Investigation*, **123**( 12), 5389- 400. <https://doi.org/10.1172/JCI70317>

Lananna, B. V., Nadarajah, C. J., Izumo, M., Cedeño, M. R., Xiong, D. D., Dimitry, J., … Musiek, E. S. (2018). Cell-autonomous regulation of astrocyte activation by the circadian clock protein BMAL1. *Cell Reports*, **25**( 1), 1- 9e5. <https://doi.org/0.1016/j.celrep.2018.09.015>

Lamia, K. A., Storch, K. F., & Weitz, C. J. (2008). Physiological significance of a peripheral tissue circadian clock. *Proceedings of the National Academy of Sciences of the United States of America*, **105**( 39), 15172- 7. <https://doi.org/10.1073/pnas.0806717105>

Rudic, R. D., McNamara, P., Curtis, A. M., Boston, R. C., Pa, S., Hogenesch, J. B., & Fitzgerald, G..A. (2004). BMAL1 and CLOCK, two essential components of the circadian clock, are involved in glucose homeostasis. *PLoS Biology*, **2**( 11), e377. <https://doi.org/10.1371/journal.pbio.0020377>

Shi, S. Q., Ansari, T. S., McGuinness, O. P., Wasserman, D. H., & Johnson, C. H. (2006). Circadian disruption leads to insulin resistance and obesity. *Current Biology*, **23**( 5), 372- 381. <https://doi.org/10.1016/j.cub.2013.01.048>

**Table S2** Specificity and efficiency of inducible GLAST-Cre-mediated recombination

| **Specificity (%/Section) Hypothalamus (%)** | | | |
| --- | --- | --- | --- |
|  | Control | *Bmal1cKO* | p Value |
| % GFAP/TdT | 53.7 ± 2.9 (n = 8) | 40.2 ± 2.9 (n = 8) | 0.006 |
| %S100β/TdT | 58.9 ± 4.6 (n = 5) | 32.5 ± 3.2 (n = 7) | 0.003 |
| **Efficiency (%/Section) Hypothalamus (%)** | | | |
|  | Control | *Bmal1cKO* | p Value |
| % TdT/GFAP | 70.9 ±3.3 (n = 8) | 51.4 ± 3.1 (n = 5) | 0.008 |
| % TdT/S100β | 50.2 ± 1.9 (n = 5) | 27.25 ± 3.2 (n = 7) | 0.015 |

Recombination in the ARC nucleus of *Glast-Cre-Td-Tomato* (control) or *Bmal1cKO-Td-Tomato* animals, two months after TM treatment.


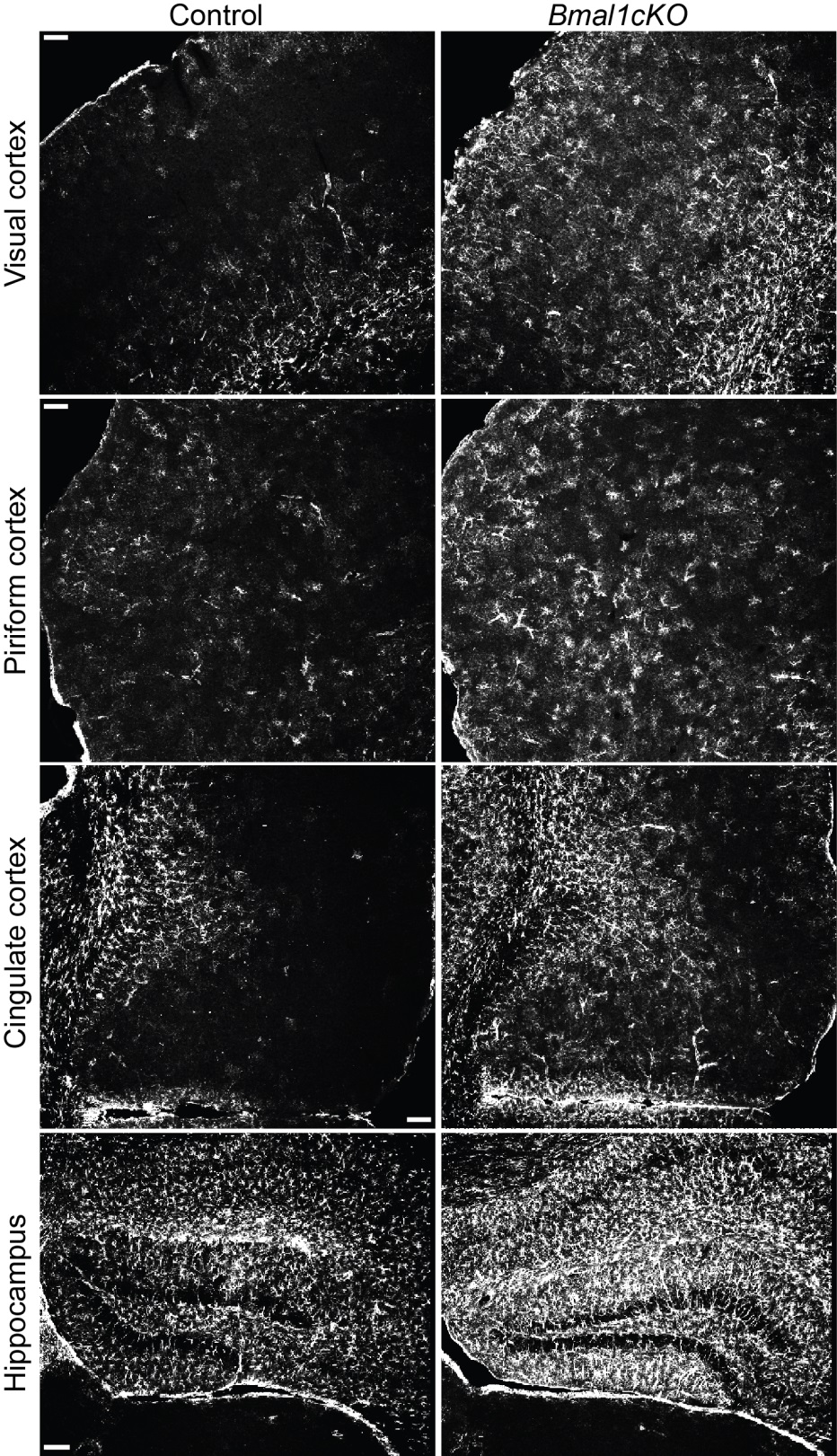


**Figure S1**

Representative micrographs of GFAP immunostaining in control and *Bmal1cKO* mice. Representative micrographs of GFAP immunostaining in the visual, piriform and cingulate cortex as well as in the hippocampus of control and *Bmal1cKO* mice four months after TM treatment. Scale bar, 100 μm.


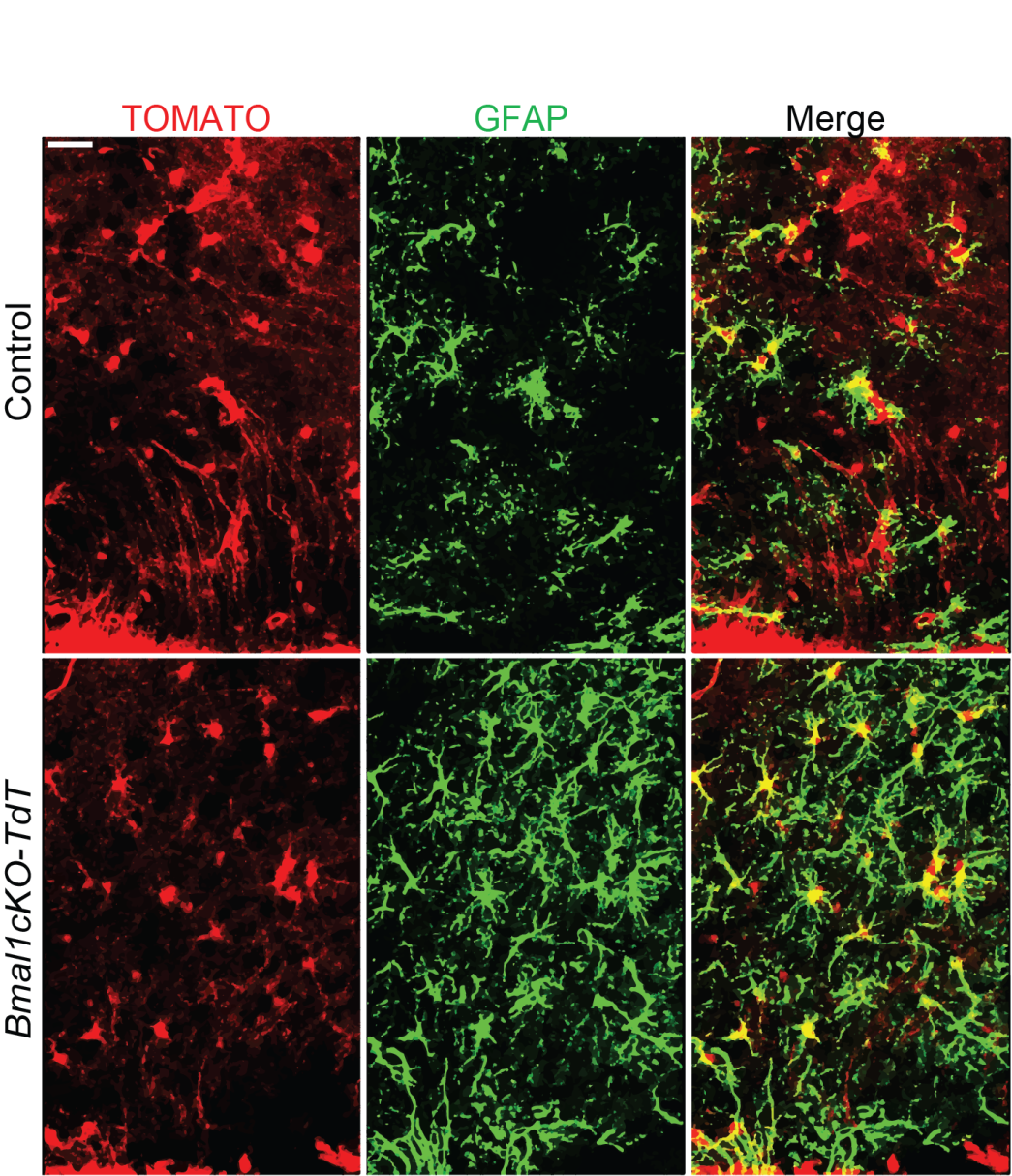


**Figure S2**

GFAP and TOMATO co-localization in the ARC nucleus of control and *Bmal1cKO* animals. Representative micrographs of TOMATO (red) and GFAP (green) immunostaining in the ARC nucleus of control (*Glast-Cre-Td-Tomato*) and *Bmal1cKO-Td-Tomato* animals two months after TM treatment. Scale bar, 25 μm.

**
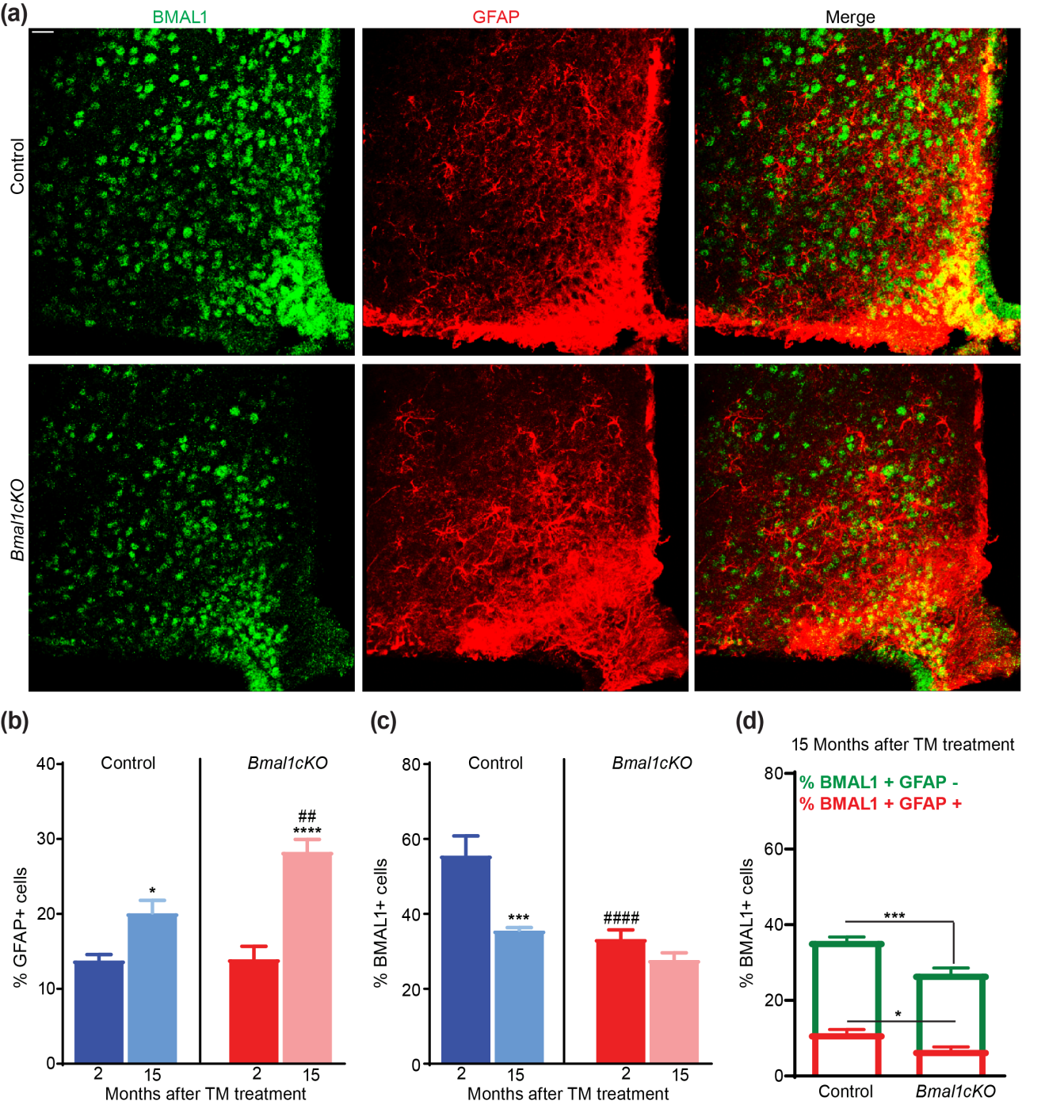
**

**Figure S3**

BMAL1 deletion in ARC astrocytes at 15 months after TM treatment. **(a)** Representative micrographs of GFAP and BMAL1 immunostaining in the ARC of control and *Bmal1cKOs* after 15 months of TM treatment. Scale bar, 25 μm. **(b)** Quantification of the percentage of GFAP-positive cells in the ARC nucleus of control and *Bmal1cKO* mice two and 15 months after TM. Data are represented as mean ± SEM (n=4, two-way ANOVA, *p<0.05 and ****p<0.0001 versus two months treated-animals; ##p<0.01 versus controls). **(b)** Quantification of the percentage of BMAL1-positive cells in the ARC nucleus of control and *Bmal1cKO* mice two and 15 months after TM. Data are represented as mean ± SEM (n=4, two-way ANOVA, ***p<0.001 versus two months treated-animals; ####p<0.0001 versus controls). **(c)** Quantification of the percentage of BMAL1-positive cells in the population of GFAP positive (red) or negative (green) cells in the ARC nucleus of control and *Bmal1cKO* mice 15 months after TM. Data are represented as mean ± SEM (n=4, paired t-test, *p<0.05 and ***p<0.001 versus control animals).


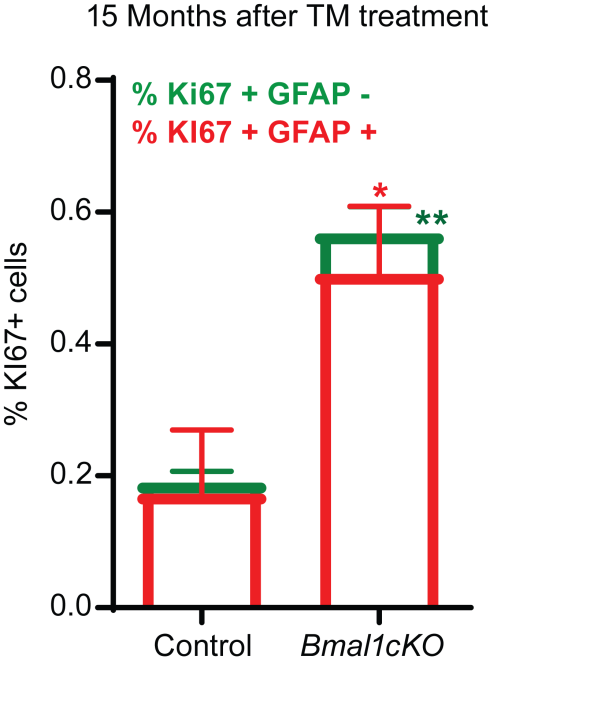


**Figure S4**

Quantification of KI67 positive astrocytes in the ARC of control and mutants at 15 months after TM treatment. An increase of KI67-positive cells was observed in the population of GFAP-positive (red, paired t-test, *p<0.05) and GFAP-negative cells (green, paired t-test, **p<0.01 versus control animals) of *Bmal1cKO* mice compared with control animals at 15 months after TM treatment.


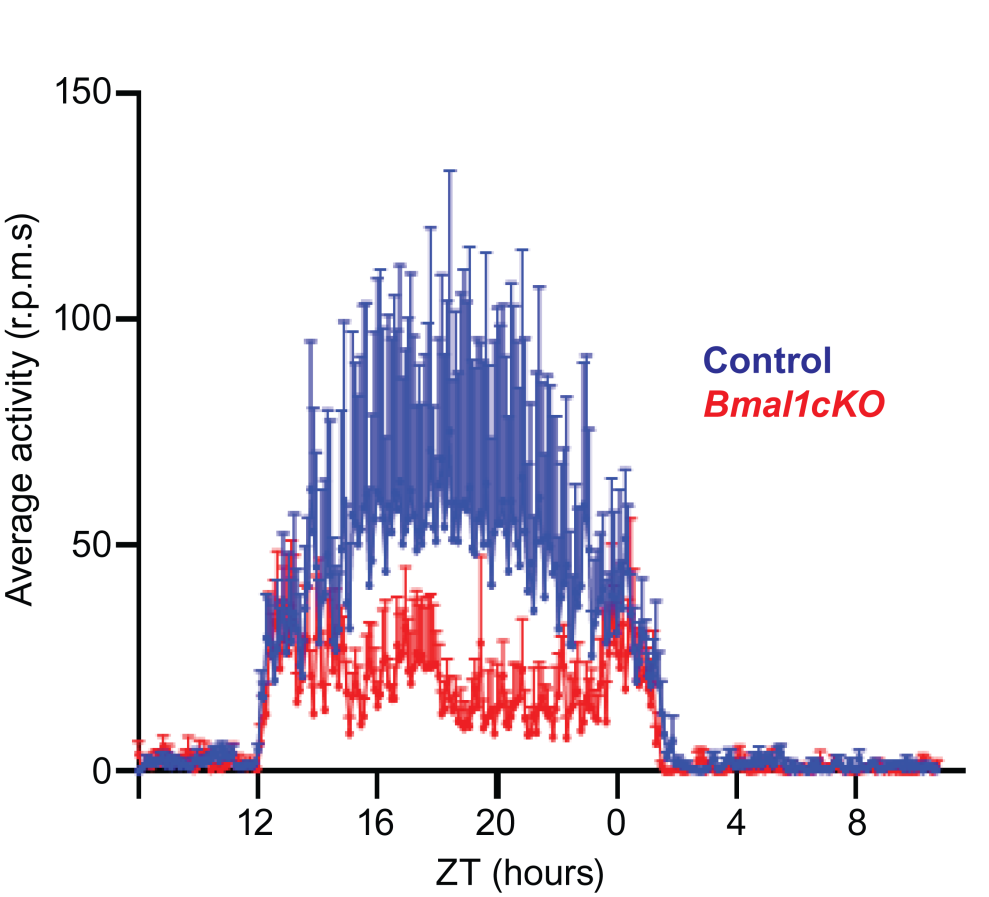


**Figure S5**

Daily activity of control and Bmal1cKO mice 15 months after TM treatment. Activity waveforms for control (n=6) and *Bmal1cKO* (n=5) mice, 15 months after TM treatment, in 12h: 12h light-dark cycles. Activity counts are expressed as the average amount of activity in five minute bins. Data plotted is given in ZT, such that ZT0 = lights on. The value expresses the means + SEM.
